# A Bacterial Toxin Perturbs Intracellular Amino Acid Balance To Induce Persistence

**DOI:** 10.1128/mBio.03020-20

**Published:** 2021-02-23

**Authors:** Xiaofeng Zhou, Michael R. Eckart, Lucy Shapiro

**Affiliations:** a School of Agriculture, Sun Yat-sen University, Shenzhen, China; b Department of Developmental Biology, Stanford University School of Medicine, Stanford, California, USA; c Stanford Protein and Nucleic Acid Facility, Stanford University School of Medicine, Stanford, California, USA; d Chan Zuckerberg Biohub, San Francisco, California, USA; Rutgers University

**Keywords:** *Caulobacter crescentus*, amino acid balance, bacterial toxin, persistence, stringent response

## Abstract

Bacterial cells utilize toxin-antitoxin systems to inhibit self-reproduction, while maintaining viability, when faced with environmental challenges. The activation of the toxin is often coupled to the induction of cellular response pathways, such as the stringent response, in response to multiple stress conditions. Under these conditions, the cell enters a quiescent state referred to as dormancy or persistence. How toxin activation triggers persistence and induces a systemic stress response in the alphaproteobacteria remains unclear. Here, we report that in *Caulobacter*, a *hipA2*-encoded bacterial toxin contributes to bacterial persistence by manipulating intracellular amino acid balance. HipA2 is a serine/threonine kinase that deactivates tryptophanyl-tRNA synthetase by phosphorylation, leading to stalled protein synthesis and the accumulation of free tryptophan. An increased level of tryptophan allosterically activates the adenylyltransferase activity of GlnE that, in turn, deactivates glutamine synthetase GlnA by adenylylation. The inactivation of GlnA promotes the deprivation of glutamine in the cell, which triggers a stringent response. By screening 69 stress conditions, we find that HipBA2 responds to multiple stress signals through the proteolysis of HipB2 antitoxin by the Lon protease and the release of active HipA2 kinase, revealing a molecular mechanism that allows disparate stress conditions to be sensed and funneled into a single response pathway.

## INTRODUCTION

Bacteria are often faced with environmental challenges. A subpopulation of cells, known as dormant cells or persisters, can stochastically or responsively enter a physiologically quiescent state to enable survival under adverse conditions (1). Phenotypic characteristics of persisters are arrested growth and inactive metabolism, which offer enhanced tolerance to challenging environmental cues (2). Persistence is noninheritable, and persisters are able to revert to wild-type cells upon removal of stress conditions (3). Bacterial persistence is recognized as one of the major mechanisms rendering resistance to antibiotics and relapsing infections during the treatment of infectious disease (2, 4).

**FIG 1:**
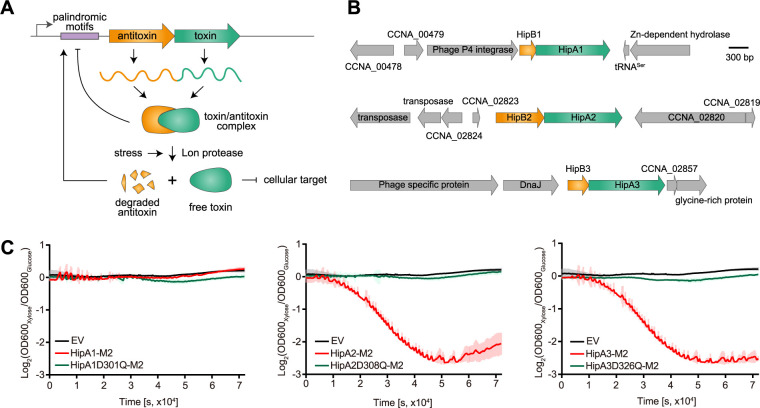
*Caulobacter* encodes three HipBA modules. (A) Schematic view of a typical type II toxin-antitoxin system. (B) Gene organization of three Caulobacter crescentus HipBA modules. (C) Relative growth (xylose versus glucose) of *Caulobacter* strains bearing chromosomal copies of *hipA* genes tagged with M2 and their kinase-dead mutants as the only copies of the *hipA* genes, transcribed from a xylose-inducible promoter, in PYE medium with either xylose or glucose. The OD was measured on a plate reader. The OD value was recorded every 5 min for 24 h. EV denotes empty vector. Mean and SD from four biological replicates are plotted.

Although persistence is a noninherited transient state of the bacterial life cycle, signaling pathways that enable bacterial persistence and the mechanisms that control persister formation are genetically encoded (4–6). A growing body of evidence suggests that bacterial toxin-antitoxin (TA) systems are linked to persister formation either directly or indirectly (7–9). A TA system is a genetic module cotranscribed from a small operon that usually consists of two gene products (Fig. 1A), a toxin that causes growth arrest by targeting a vital cellular component and a cognate antitoxin which neutralizes the toxin under normal growth conditions (10, 11). TA modules are classified based on the nature of the antitoxin and its working mechanism (11). The antitoxin is usually degraded by cellular proteases in response to various types of environmental signals (12). This posttranslational regulation enables the toxin to be released from the toxin-antitoxin protein complex, resulting in cellular intoxication (10). Moyed and Bertrand provided the first experimental evidence linking a TA module to persistence in Escherichia coli by identifying a mutation in a HipBA module (13). HipBA belongs to the type II TA system, in which antitoxins directly bind and neutralize toxins, and is encoded in a broad range of bacterial species (14). The *hipA* gene encodes a eukaryote-like serine-threonine kinase that phosphorylates tRNA-synthetase to inhibit protein translation (8, 9), while HipB antitoxin neutralizes HipA toxicity by forming a heterotetrameric protein complex, which is also capable of binding to the promoter region of HipBA module to repress its transcription (15).

Rather than inducing cell dormancy, the prolonged inhibition of protein synthesis usually leads to cell death (16). Therefore, HipA-mediated persistence is often coupled to the activation of the stringent response (8, 9), which relies on the production of guanosine tetraphosphate and guanosine pentaphosphate [collectively referred to as (p)ppGpp] as the ubiquitous “alarmone” that binds to essential regulatory proteins to reprogram cellular physiology (17, 18). Although the stringent response is a broadly conserved mechanism for bacteria to adapt to nutrient starvation and other environmental challenges, the molecular details activating the stringent response appear to vary from bacterium to bacterium (19). In E. coli, (p)ppGpp homeostasis is dependent on two enzymes, the (p)ppGpp synthetase RelA and the bifunctional (p)ppGpp synthetase/hydrolase SpoT. In alphaproteobacteria, however, RelA is often absent and the (p)ppGpp turnover is strictly dependent on a single RelA/SpoT homologue, named SpoT due to its bifunctional activity (20, 21). In Caulobacter crescentus, the activation of SpoT relies on nitrogen starvation by a nitrogen-related phosphotransferase system (PTS^Ntr^) that consists of three enzymes (22). The first enzyme, EI^Ntr^, detects glutamine deprivation by autophosphorylation (22). This signal is transduced to two other components, HPr and EII^Ntr^, which physically bind SpoT to regulate its activity (22). This pathway is conserved in alphaproteobacteria in response to nitrogen availability in the oligotrophic environment (23). Although HipA-mediated (p)ppGpp accumulation and bacterial persistence are well characterized in E. coli, that is, RelA protein is activated by stalled ribosomes, little is yet known about how HipA toxin induces (p)ppGpp production in alphaproteobacteria lacking RelA protein.

In this study, we discovered that the HipA2 toxin in *Caulobacter* induces bacterial persistence and (p)ppGpp accumulation by disturbing intracellular tryptophan-glutamine balance. HipA2 phosphorylates tryptophanyl-tRNA synthetase TrpS, which stimulates adenylylation of glutamine synthetase GlnA by the allosteric regulation of GlnE and GlnD. Adenylylated GlnA is no longer active for glutamine synthesis, thereby resulting in glutamine deprivation. The conserved PTS^Ntr^ pathway, sensing glutamine deprivation, activates SpoT and (p)ppGpp accumulation. We screened 69 stress conditions and found that nutrient starvation and physical/chemical stress triggered the activation of HipA2 toxins by Lon-mediated proteolysis of HipB2 antitoxin. Our results reveal a unique mechanism by which bacterial cells utilize a toxin protein to sense disparate stress conditions which are in turn translated into nitrogen starvation for environmental adaption.

## RESULTS

### *Caulobacter* HipBA2 and HipBA3 function as toxin-antitoxin systems.

BLAST analysis and a paralog search revealed that C. crescentus encodes three potential HipBA toxin-antitoxin modules (HipBA1 CCNA_00481-CCNA_00482, HipBA2 CCNA_02822-CCNA_02821, and HipBA3 CCNA_02859-CCNA_02858) (Fig. 1B). To test whether the three HipA proteins function as bona fide toxins in C. crescentus, we individually expressed three *hipA* genes as well as each kinase-dead mutant, which lacks kinase activity (24), fused with an M2 tag under the control of the xylose promoter (integrated at *xylX* locus) in a strain background lacking all three HipBA modules (*ΔhipBA1BA2BA3*). The *ΔhipBA1BA2BA3* was used as a background to avoid cross talk interference between each toxin gene. Cells expressing *hipA2* or *hipA3* exhibited significant growth arrest that is similar to a previous report (25), while cells harboring an empty vector or expressing kinase-dead *hipA* mutants maintained the normal growth (Fig. 1C). It is noteworthy that expression of *hipA1* failed to confer the growth arrest phenotype under these growth conditions, suggesting that HipA1 may not function as a toxin in *Caulobacter* (Fig. 1C). To confirm this observation, we switched our inducible expression system from the xylose promoter to the vanillate promoter and observed the same growth phenotype for the three HipA proteins with a hemagglutinin (HA) tag at their C terminus (see Fig. S1A in the supplemental material). Expression of each of the HipA genes was confirmed using protein immunoblots (Fig. S1B). We ruled out the possibility of suppressor mutation rescue for strains expressing the *hipA1* gene by full-genome sequencing (see the supplemental material). These experiments suggest that HipBA2 and HipBA3 encode toxin-antitoxin systems in *Caulobacter*.

10.1128/mBio.03020-20.2 FIG S1 Under conditions tested, HipA1 is not a toxin. (A) Bacterial growth curves in PYE medium supplemented with 0.5 mM vanillate. Wild-type strains harboring the indicated toxins were grown in PYE for 3 h followed by the addition of vanillate inducers. Expressions of HipA2 and HipA3 caused growth arrest. Mean and SD from four biological replicates are plotted. (B) Verification of protein expressions in Fig. 1C and Fig. S1A by immunoblotting. Download FIG S1, EPS file, 1.1 MB. Copyright © 2021 Zhou et al. 2021 Zhou et al. https://creativecommons.org/licenses/by/4.0/ This content is distributed under the terms of the Creative Commons Attribution 4.0 International license.

### HipA2 phosphorylates tryptophanyl-tRNA synthetase.

**FIG 2:**
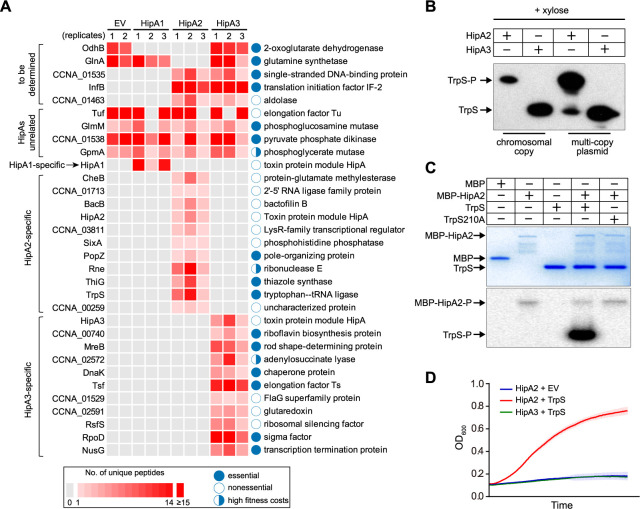
HipA2 phosphorylates tryptophanyl-tRNA synthetase. (A) Phosphoproteomic analysis of *Caulobacter* cells expressing individual HipA toxins under the control of an inducible xylose promoter. Cells containing an empty vector serve as a negative control to rule out HipA-independent phosphorylation events. Three replicates were assayed for strains expressing the indicated HipA proteins, and two replicates were included for the empty vector (EV) control samples. The number of identified phosphopeptides and the corresponding proteins are plotted. (B) *In vivo* phosphorylation of TrpS by HipA2. HA-tagged TrpS was natively expressed (either from a single copy on the chromosome or from a multicopy plasmid) in the *ΔhipBA1BA2BA3* mutant expressing HipA2 or HipA3 toxin under the control of the xylose promoter. Whole-cell lysates were analyzed by Phos-tag SDS-PAGE and immunoblotting with anti-HA antibody. (C) *In vitro* phosphorylation of TrpS by HipA2. Purified TrpS or a TrpS210A mutant was incubated with MBP-HipA2 and γ-^32^P-labeled ATP for 45 min. Samples were analyzed by SDS-PAGE (top) and autoradiography (bottom). (D) Growth curves of wild-type cells coexpressing the indicated HipA toxin on the chromosome and TrpS on a multicopy plasmid. Overexpression of TrpS restores the HipA2-induced growth defect. Mean and SD from four biological replicates are plotted.

HipA toxin encodes a serine/threonine kinase, by which essential cellular components are often phosphorylated. To biochemically identify the *Caulobacter* HipA targets, we conducted a genome-wide comparative phosphoproteomic analysis of cells conditionally activating HipA toxin in the *ΔhipBA1BA2BA3* mutant using mass spectrometry (MS). The growth of this mutant was similar to the wild type, suggesting that all three HipBA modules are not required for growth under normal conditions (Fig. S2A). Each *hipA* gene was individually expressed from a *P_xyl_* promoter in the *ΔhipBA1BA2BA3* background so that the toxin gene could be conditionally expressed to allow cell growth and to prevent suppressor mutations of potential HipA targets (Fig. S2B). Our phosphoproteomic analysis includes three biological replicates for strains expressing each toxin and two biological replicates for the control strain harboring an empty vector. Identified phosphoproteins for all replicates were listed in Table S1 and plotted as a Venn diagram in Fig. S2C. A protein is considered a potential HipA target if it exhibits the following three criteria: (i) it is not phosphorylated in either replicate of the empty vector control sample, (ii) it must be identified as a phosphoprotein in all three replicates of samples expressing any one of the three HipA proteins but not samples expressing any of the other two HipA proteins, and (iii) the phosphorylation site must be identical among all three replicates. Imposing these criteria yielded 11 potential targets for HipA2 and a different set of 11 targets for HipA3 (Fig. 2A). Notably, in the case of HipA1, we found that only its autophosphorylation fulfilled the above criteria, which is consistent with the notion that HipA1 is not a toxin in *Caulobacter* (Fig. 2A).

10.1128/mBio.03020-20.3 FIG S2 Identification of targets for *Caulobacter* HipAs. (A) Bacterial growth curves of the wildstype and *ΔhipBA1BA2BA3* strains. (B) Experimental design of phosphoproteomic analysis used to identify HipA targets. (C) Venn diagram displays the number of identified unique phosphopeptides for the indicated toxins. The plotted numbers are the sum of three independent biological replicates. (D) Verification of *in vivo* phosphorylation of candidate targets by HipA2 using Phos-tag SDS-PAGE. (E) Verification of *in vivo* phosphorylation of candidate targets by HipA3 using Phos-tag SDS-PAGE. Asterisk indicates nonspecific or cleaved band. (F) *In vitro* autophosphorylation of *Caulobacter* HipA toxins analyzed by SDS-PAGE (top) and autoradiography (bottom) using purified HipA proteins incubated with γ-^32^P-labeled ATP. (G) Schematic view of conserved operator sites in the promotor region of HipBA modules in *Caulobacter*. Download FIG S2, EPS file, 1.0 MB. Copyright © 2021 Zhou et al. 2021 Zhou et al. https://creativecommons.org/licenses/by/4.0/ This content is distributed under the terms of the Creative Commons Attribution 4.0 International license.

10.1128/mBio.03020-20.8 TABLE S1 Phosphorylated proteins and peptides identified by LC-MS. Download Table S1, XLSX file, 0.03 MB. Copyright © 2021 Zhou et al. 2021 Zhou et al. https://creativecommons.org/licenses/by/4.0/ This content is distributed under the terms of the Creative Commons Attribution 4.0 International license.

To verify the phosphorylation of potential targets found by mass spectrometry, we used both Phos-tag gels of cell extracts and *in vitro* protein kinase assays. For the proteins apparently phosphorylated by HipA2, we focused on PopZ, ThiG, and TrpS as they were shown to be essential proteins in *Caulobacter* grown in rich PYE medium (26). We observed significantly retarded migration of TrpS, but not PopZ and ThiG, in cells expressing HipA2 (Fig. 2B and Fig. S2D). This retardation was not seen in cells expressing HipA3, demonstrating that TrpS is one target of HipA2. TrpS S210 was identified as a phosphorylated residue by our mass spectrometry analysis. To verify this phosphorylation site, we performed *in vitro* kinase assays using purified TrpS, TrpS210A mutant, and MBP-HipA2 proteins. The phosphorylation of TrpS by MBP-HipA2 was detected while the TrpS210A mutant failed to be phosphorylated by MBP-HipA2, suggesting that serine 210 in TrpS is an orphan residue upon HipA2-mediated phosphorylation (Fig. 2C). Overexpression of TrpS on a multicopy plasmid restored the growth arrest caused by HipA2 expression (Fig. 2D). Consistently, we detected both phosphorylated TrpS and unphosphorylated TrpS in this strain (Fig. 2B). In our studies, we failed to detect GltX phosphorylation by HipA2 (Fig. S2E), as previously reported (25). Cumulatively, our results suggest that HipA2 phosphorylates TrpS to render the growth arrest phenotype. For the targets of HipA3 protein, six essential proteins were tested for *in vivo* phosphorylation. However, none of them showed positive results (Fig. S2E). HipA3 is likely a noncanonical HipA family toxin (see Discussion). Here, we have focused on the mechanism of HipA2 toxin function.

### (p)ppGpp accumulation contributes to swarmer cell viability during HipA2-induced growth arrest.

**FIG 3:**
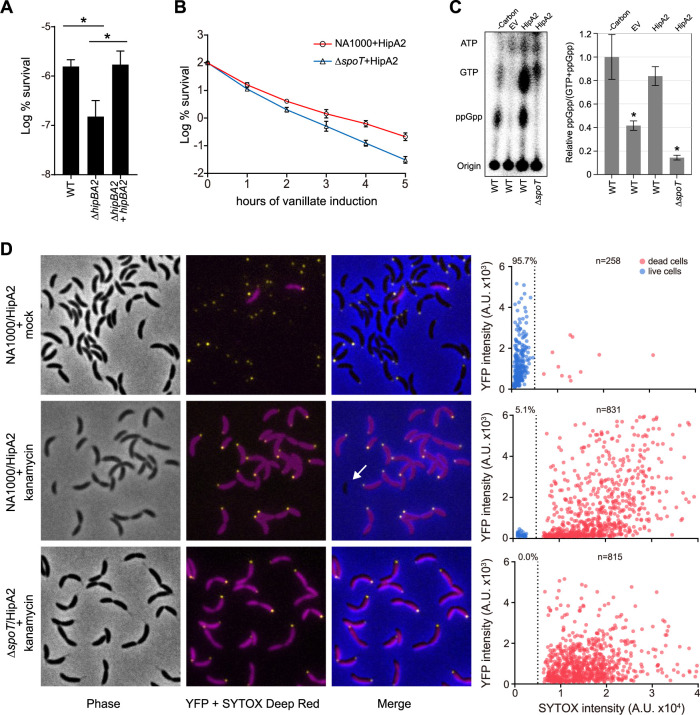
(p)ppGpp accumulation contributes to cell viability in swarmer cells during HipA2-induced growth arrest. (A) The indicated strains were grown to late exponential phase from approximately the same starting density. Cultures were then challenged with kanamycin (5 μg/ml) for 4 h. Results are shown as percent survival rate by comparison to untreated cells prior to the addition of antibiotic. Mean and SD from at least two independent experiments performed with three biological replicates (*n *= 6) are plotted. An asterisk indicates a significant difference (*P* < 0.05) by two-tailed Student’s *t* test. (B) The wild-type and *ΔspoT* strains harboring an extra copy of HA-HipA2 on the chromosome at the *vanR* locus were grown to the late exponential phase from approximately the same starting density. HipA2 expression was induced by the addition of 0.5 mM vanillate. Results show the percentage of survival at each indicated time point of vanillate treatment (log scale). Mean and SD from at least two independent experiments performed with three biological replicates (*n *= 6) are plotted. (C) Expression of HipA2 stimulates (p)ppGpp accumulation. Intracellular levels of (p)ppGpp extracted from the wild-type and *ΔspoT* strains expressing HipA2 toxin were detected by TLC. Mean and SD are plotted (*n *= 4). An asterisk indicates a significant difference (*P* < 0.05) by two-tailed Student’s *t* test in comparison with carbon starvation-treated wild-type cells. (D) SYTOX Deep Red staining reveals the persistence trait of swarmer cells in a (p)ppGpp-dependent manner. The indicated strains expressing DivJ-YFP were grown in PYE to the exponential phase. Cultures were supplemented with vanillate to induce HipA2 expression for 3 h and then challenged with kanamycin (5 μg/ml) for 4 h followed by SYTOX Deep Red staining according to manufacturer’s instructions. The arbitrary fluorescent intensities of YFP and SYTOX Deep Red are plotted.

HipBA toxin-antitoxin modules were shown to induce bacterial persistence in E. coli (8, 9). In *Caulobacter*, we observed a typical biphasic killing curve when cells were exposed to bactericidal antibiotics that are lethal to *Caulobacter* (Fig. S3A). Most of the susceptible population was killed rapidly within 2 h of exposure, while the persister cells were revealed after 4 h of exposure where the curve is in a plateau phase (Fig. S3A). A comparison of the survival rates of wild-type and *ΔhipBA2* cells after 4 h of exposure to a lethal concentration of gentamicin revealed that the survival rate of *ΔhipBA2* cells is significantly lower than that of wild-type cells (Fig. 3A). As expected, if this was due to the absence of the toxin-antitoxin system, the loss of viability was restored by the addition of plasmid carrying the entire *hipBA2* operon (Fig. 3A).

10.1128/mBio.03020-20.4 FIG S3 *Caulobacter* swarmer cells form persisters. (A) Biphasic killing curves exhibit a high or low rate of killing by kanamycin (5 μg/ml) and gentamycin (1 μg/ml). (B) Survival curves of swarmer cells isolated from the indicated strains. The wild-type and *ΔspoT* strains were grown to the late exponential phase from approximately the same starting density. The expressions of HipA2 were induced by the addition of 0.5 mM vanillate for 3 h. Swarmer cells were isolated and resuspended in PYE supplemented with 0.5 mM vanillate for synchronization. Cells were drawn at each indicated time point, washed with PBS buffer, and plated for CFU enumeration. Mean and SD from at least two independent experiments performed with three biological replicates (*n *= 6) are plotted. Download FIG S3, EPS file, 1.2 MB. Copyright © 2021 Zhou et al. 2021 Zhou et al. https://creativecommons.org/licenses/by/4.0/ This content is distributed under the terms of the Creative Commons Attribution 4.0 International license.

Persistence in E. coli was shown to be dependent on a toxin-antitoxin HipBA module by a mechanism which activates a stringent response via the accumulation of the alarmone (p)ppGpp (8, 9, 18). The turnover of (p)ppGpp in *Caulobacter* is strictly controlled by a bifunctional synthase/hydrolase, SpoT (21). To test whether (p)ppGpp is involved in HipA2-induced persistence in *Caulobacter*, we measured the survival rates of cells expressing HipA2 in the wild-type strain and *ΔspoT* mutant. Cells lacking *spoT* showed decreased viability when expressing the HipA2 protein from an inducible vanillate promoter (Fig. 3B). We confirmed the accumulation of (p)ppGpp in cells expressing induced levels of HipA2 by thin-layer chromatography (TLC) (Fig. 3C). Together, these results show that HipA2 is associated with persister formation in *Caulobacter* and that (p)ppGpp contributes to cell viability when bacterial growth is arrested by the expression of HipA2 toxin.

It was reported that *Caulobacter* swarmer cells (motile progeny cells) produce (p)ppGpp at a higher rate than stalked cells (sessile progeny cells) upon starvation (27) and that (p)ppGpp accumulation decreases the growth rate and the swarmer-to-stalked cell transition (28). It is tempting to speculate that swarmer cells could be the repository of the persister population due to the accumulation of (p)ppGpp when HipA2 is activated. To test this hypothesis, we performed SYTOX Deep Red staining for wild-type and *ΔspoT* cells expressing HipA2 toxin followed by 4 h of exposure to a lethal concentration of kanamycin. To distinguish swarmer cells from other cell types, both stains contained a *divJ-yfp* allele instead of the native *divJ*. The histidine kinase DivJ localizes specifically to the stalked pole during the swarmer-to-stalked cell transition (29, 30). Fluorescently labeled DivJ serves as a marker for stalked and predivisional cells and distinguishes them from swarmer cells. In the absence of exposure to kanamycin, nearly 96% of cells expressing induced levels of HipA2 were viable (Fig. 3D). In contrast, only 5.1% of cells expressing HipA2 remained alive upon exposure to kanamycin for 4 h, all of which lacked DivJ-YFP (yellow fluorescent protein) signal, suggesting that they were swarmer cells (Fig. 3D). Under similar conditions, we did not observe any viable cells from the *ΔspoT* strain (Fig. 3D). In addition, the isolated wild-type swarmer cells were significantly more resistant to antibiotic exposure than *ΔspoT* swarmer cells upon HipA2 activation (Fig. S3B). These results suggest SpoT-controlled (p)ppGpp turnover in swarmer cells contributes to *Caulobacter* persistence.

### HipA2 triggers an imbalance of the intracellular tryptophan and glutamine by stimulating GlnA adenylylation.

**FIG 4:**
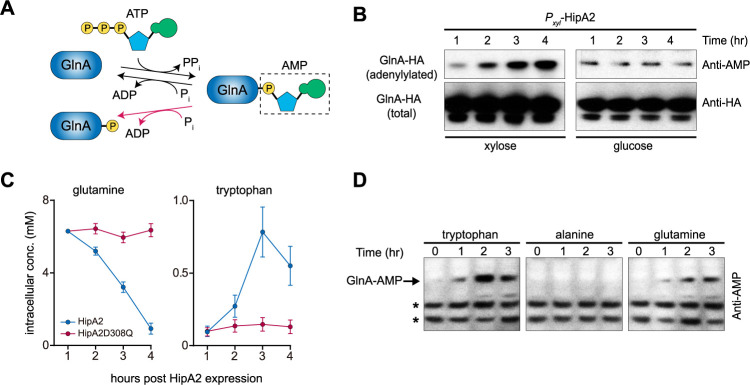
HipA2 triggers an imbalance of intracellular free tryptophan and glutamine by stimulating GlnA adenylylation. (A) Schematic representation of GlnA adenylylation and deadenylylation. GlnA deadenylylation can be achieved by both hydrolysis (black arrow) and phosphorolysis (red arrow). (B) Stimulation of GlnA adenylylation by HipA2 analyzed by immunoprecipitation. Cells expressing HA-GlnA controlled by its native promoter and HipA2 controlled by the inducible xylose promoter were grown in PYE + glucose to exponential phase. Cells were collected, washed, and resuspended in PYE + xylose or PYE + glucose for induction or repression of HipA2 expression, respectively. Samples were collected at each indicated time point for immunoprecipitation using anti-HA magnetic beads and analyzed by SDS-PAGE and immunoprecipitation with either anti-AMP or anti-HA antibody. (C) Quantification of intracellular levels of free tryptophan and glutamine upon HipA2 activation. Strains harboring a chromosomal copy of *P_xyl_-hipA2* or *P_xyl_-hipA2D308Q* (kinase-dead mutant) were grown in PYE + glucose to the exponential phase. Cells were collected, washed, and resuspended in PYE + xylose for HipA2 induction. Samples were collected at each indicated time point for free amino acid quantification, as described in Materials and Methods. (D) Comparison of GlnA adenylylation levels upon exposure to exogenous amino acids. Wild-type Caulobacter crescentus was grown in M2G medium to the exponential phase, and the culture was divided into three equal aliquots. The indicated amino acids were added to each aliquot, and cells were collected each hour for a period of 3 h. Lysates were prepared from each sample and analyzed by SDS-PAGE and immunoblotting with anti-AMP antibody. Due to the limited number of proteins that are adenylylated in *Caulobacter* (indicated by asterisks), we were able to compare levels of GlnA adenylylation in response to amino acid treatment.

Unlike E. coli, in which (p)ppGpp accumulation-induced HipA is dependent on RelA (8, 9), *Caulobacter* lacks homologous RelA and the (p)ppGpp turnover is strictly controlled by SpoT, a single bifunctional synthetase-hydrolase enzyme (27). SpoT synthetase activity is activated by a nitrogen-related phosphotransferase system upon glutamine deprivation, whose regulation is dependent on a nitrogen regulatory circuit (22, 23). Mass spectrometry analysis revealed dephosphorylation of GlnA tyrosine 398 upon HipA2 expression (Fig. 2A and Fig. S4A), which was verified *in vivo* by Phos-tag SDS-PAGE (Fig. S4B). Tyrosine 398 of GlnA was robustly phosphorylated in cells expressing HipA1 and HipA3, as well as in control cells (Fig. 2A). Although GlnA Y398 phosphorylation has not yet been reported for *Caulobacter*, multiple studies have demonstrated that this residue undergoes adenylylation and deadenylylation in a nitrogen-rich and -poor environment, respectively (31, 32). To test the possibility that GlnA phosphorylation could be a deadenylylation by-product generated by phosphorolysis rather than hydrolysis (Fig. 4A), HA-tagged GlnA protein was immunoprecipitated in a strain expressing xylose-inducible HipA2. Protein samples were subjected to immunoblot analysis against either anti-HA or anti-AMPylated tyrosine antibody. The intracellular level of adenylylated GlnA gradually increased over the course of xylose induction of HipA2 expression (Fig. 4B). Conversely, the intracellular levels of adenylylated GlnA remained constant when HipA2 expression was inhibited by the addition of glucose (Fig. 4B).

10.1128/mBio.03020-20.5 FIG S4 Phosphorylation of GlnA in *Caulobacter*. (A) Mass spectrum of GlnA phosphorylation at tyrosine 398. The spectrum was pulled from one of the control samples analyzed by mass spectrometry. (B) Verification of GlnA dephosphorylation by the expression of HipA2 using Phos-tag SDS-PAGE. Download FIG S4, EPS file, 0.7 MB. Copyright © 2021 Zhou et al. 2021 Zhou et al. https://creativecommons.org/licenses/by/4.0/ This content is distributed under the terms of the Creative Commons Attribution 4.0 International license.

Because HipA2 expression resulted in the adenylylation of GlnA (Fig. 4B), which would inhibit glutamine synthesis (33), and in the phosphorylation of TrpS (Fig. 2B), which would block the consumption of free tryptophan (9), we determined the intracellular levels of free glutamine and tryptophan. We observed decreased levels of free glutamine and increased levels of free tryptophan over the course of HipA2 induction (Fig. 4C). To determine if stimulation of GlnA adenylylation and glutamine deprivation can be ascribed to the accumulation of intracellular free tryptophan, total lysates of cells treated with tryptophan (1 mM), alanine (1 mM), and glutamine (10 mM) were assayed for GlnA adenylylation by SDS-PAGE gel immunoblotting. Our results showed that both tryptophan and glutamine were able to trigger GlnA adenylylation but alanine was not (Fig. 4D). Taken together, these results suggest that expression of HipA2 leads to the accumulation of intracellular free tryptophan, which in turn negatively regulates glutamine accumulation by stimulating GlnA adenylylation.

### Tryptophan directly binds GlnD and GlnE to stimulate (p)ppGpp accumulation.

**FIG 5:**
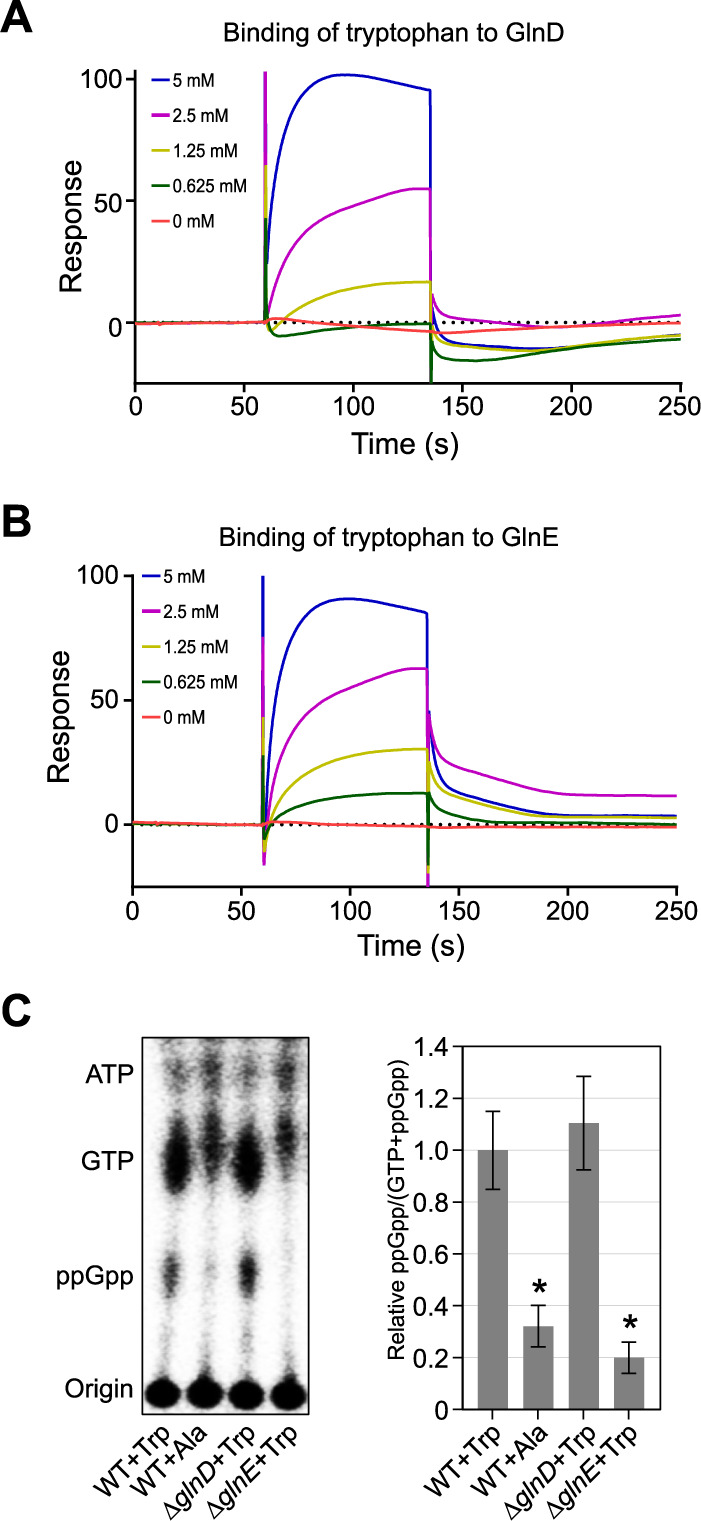
Tryptophan directly binds GlnD and GlnE to trigger (p)ppGpp accumulation. (A and B) Surface plasmon resonance (SPR) experiments show that tryptophan binds GlnD and GlnE. Purified GlnD or GlnE was tethered to the SPR chip and exposed to increasing concentrations of tryptophan. The binding period is displayed as response units plotted as function of time, followed by buffer injection to remove free tryptophan. Increasing concentrations of tryptophan used in the SPR experiments are shown. (C) Tryptophan stimulates (p)ppGpp accumulation. (Left) Intracellular levels of (p)ppGpp were detected by TLC. Alanine serves as a negative control. (Right) Relative concentrations of ppGpp as a function of tryptophan availability are shown. Mean and SD are plotted (*n *= 4). An asterisk indicates a significant difference (*P* < 0.05) by two-tailed Student’s *t* test in comparison with tryptophan-treated wild-type cells.

It is known that in E. coli the uridylyltransferase GlnD and the adenylyltransferase GlnE can directly sense the intracellular glutamine pool to adapt nitrogen metabolism (34, 35). Given our observation that a high level of free tryptophan regulates the intracellular glutamine pool by stimulating GlnA adenylylation, we hypothesized that tryptophan plays a role similar to glutamine in the allosteric regulation of GlnD and GlnE. To test this hypothesis, we performed surface plasmon resonance (SPR) binding experiments using purified recombinant fusion proteins (His tagged) to test if tryptophan directly binds to GlnD and GlnE. After immobilizing purified proteins on the SPR sensor chip, we injected increasing concentrations of tryptophan or glutamine. We observed concentration-dependent binding of tryptophan to both GlnD (Fig. 5A) and GlnE (Fig. 5B), suggesting a possible allosteric mechanism similar to that observed with glutamine (Fig. S5), albeit to a different degree. We anticipated that tryptophan might be a decoy analog of glutamine, whose intracellular concentration enabled activation of SpoT and the stringent response. To this end, we performed TLC experiments to quantify (p)ppGpp accumulation in wild-type cells treated with 1 mM l-tryptophan. In keeping with our hypothesis, exogenous supplementation with tryptophan elicited the accumulation of (p)ppGpp, which was not observed upon the addition of alanine (Fig. 5C). Moreover, tryptophan-mediated (p)ppGpp accumulation was not dependent on the uridylyltransferase GlnD but required the adenylyltransferase GlnE (Fig. 5C), implying that the regulation of GlnE alone by tryptophan was sufficient to trigger a stringent response and that GlnD activation may occur in concert with GlnE activation.

10.1128/mBio.03020-20.6 FIG S5 Glutamine binds GlnD and GlnE. (A and B) Purified GlnD (A) or GlnE (B) was tethered to the SPR chip and exposed to increasing concentrations of glutamine. The binding period is displayed as response units plotted as function of time, followed by buffer injection to remove free glutamine. Increasing concentrations of glutamine used in SPR are shown. Download FIG S5, EPS file, 1.6 MB. Copyright © 2021 Zhou et al. 2021 Zhou et al. https://creativecommons.org/licenses/by/4.0/ This content is distributed under the terms of the Creative Commons Attribution 4.0 International license.

### Multiple stress conditions activate HipA2 toxin by Lon-mediated proteolytic regulation of HipB2 antitoxin.

**FIG 6:**
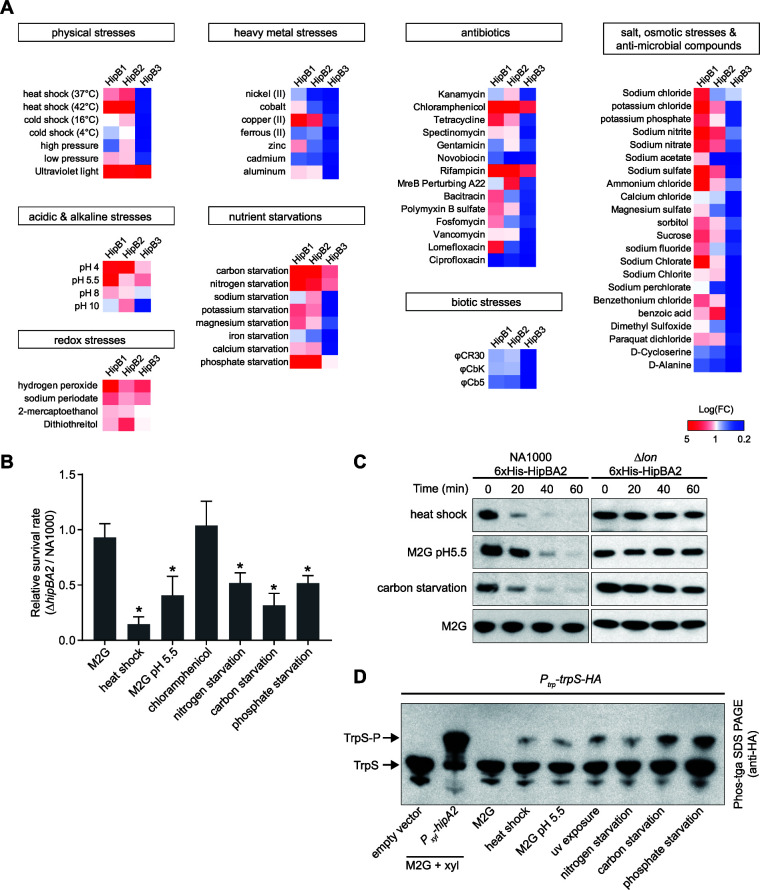
*Caulobacter* utilizes the stringent response to cope with multiple stresses by activation of HipA2 toxin. (A) Screening of environmental cues required for toxin activation in wild-type Caulobacter crescentus. Cultures were exposed to 69 different stress conditions and tested for their ability to induce upregulated transcription of *hipB* genes by qRT-PCR. The log-fold change [Log(FC)] of *hipB1*, *hipB2*, and *hipB3* transcription in strains challenged by those stresses was plotted in a heat map. The category of stress conditions used for this study is indicated, and specific conditions, agents, and antibiotics are listed in Table S2. (B) Survival rates of the Δ*hipBA2* mutant relative to the wild-type strain. Cells were grown to the exponential phase from approximately the same starting density. Cultures were then challenged with indicated stresses for 30 min (see Table S2 for detailed information) followed by the addition of kanamycin (5 μg/ml) for another 4 h. Results are shown as percent survival rate by comparison to untreated cells prior to the addition of the antibiotic. Mean and SD from one batch performed with three biological replicates (*n *= 6) are plotted. An asterisk indicates a significant difference (*P* < 0.05) by two-tailed Student’s *t* test. (C) The HipB2 protein is degraded by the Lon protease under conditions of exposure to heat shock, low pH, and carbon starvation. Wild-type (WT) and *Δlon* strains harboring a *hipBA2* operon (expressing 6×His*-hipB* and native *hipA*) on a plasmid were grown in M2G to exponential phase. Cells were then suspended in the medium conferring the indicated stresses with an additional supplement of chloramphenicol (200 μg/ml) to shut off protein synthesis. Samples were collected every 20 min, and protein levels were monitored by immunoblot assays using anti-His antibody. 6×His-*hipB* was stable in *Δlon* cells. (D) *In vivo* phosphorylation of TrpS under the challenge of various stresses. Cells expressing TrpS-HA were treated with the indicated stresses for 3 h. Whole-cell lysates were analyzed by Phos-tag SDS-PAGE and immunoblotting with anti-HA antibody. Samples containing an empty vector or expressing HipA2 served as negative and positive controls, respectively.

We screened 69 stress conditions to identify those that activate each of the HipBA toxin-antitoxin modules, so that cells could enter a physiologically quiescent state. In known type II TA systems, the antitoxin protein was shown to be degraded under specific stress conditions, which release toxin and free the TA operon promoter region bound by a heterotetrameric toxin-antitoxin protein complex (36) (Fig. 1A). We reasoned that toxin activation in *Caulobacter* could be screened by monitoring the transcription level of the paralog *hipBA* TA operons. Accordingly, we measured *hipB* mRNA levels under conditions that included physical, chemical, and biotic stresses (see the full list of stress conditions in Table S2). The expression of *hipB2* was significantly upregulated in cells treated with heat shock, acidic stress, UV exposure, and carbon, nitrogen, and phosphate starvation (Fig. 6A). Unlike E. coli, *Caulobacter hipB2* was insensitive to most of the tested antibiotics. Only chloramphenicol and rifampin were able to induce significant upregulation of *hipB2* (Fig. 6A). Assays of *hipB1* and *hipB3* transcription levels in strains exposed to these stress conditions revealed that a majority of conditions activated *hipB1* transcription despite the fact that under our growth conditions, we were unable to detect HipA1-induced growth arrest (Fig. 1C). In contrast, *hipB3* transcription was activated by only a limited number of stress conditions (Fig. 6A).

10.1128/mBio.03020-20.9 TABLE S2 The list of stress conditions tested in this study. Download Table S2, XLSX file, 0.02 MB. Copyright © 2021 Zhou et al. 2021 Zhou et al. https://creativecommons.org/licenses/by/4.0/ This content is distributed under the terms of the Creative Commons Attribution 4.0 International license.

To determine if the stress conditions that activate *hipB2* transcription in turn contribute to HipBA2-mediated persistence, we assayed bacterial survival rates of wild-type and *ΔhipBA2* strains under the six types of stress conditions shown in Fig. 6A. HipA2 contributed to bacterial survival under five of the tested conditions, while treatment with chloramphenicol did not (Fig. 6B), suggesting that chloramphenicol did not contribute to persister formation in a HipA2-dependent manner (Fig. 6B). Because the degradation of the HipB antitoxin by Lon protease in E. coli is necessary for HipA to function as a toxin (37), we measured *Caulobacter* HipB2 stabilities in wild-type and *lon* mutant strains, under conditions of heat shock, acidic stress, and carbon starvation, and found that all three conditions stimulated HipB2 instability, which was not observed in cells lacking Lon (Fig. 6C). To test if those conditions also induced TrpS phosphorylation by the liberated HipA2 toxin, we performed Phos-tag SDS-PAGE using bacterial lysates of cells under multiple stress conditions followed by protein immunoblotting. The phosphorylation of TrpS was detected under six distinct stress conditions, albeit to different degrees (Fig. 6D). Cumulatively, our data demonstrate that *Caulobacter* utilizes HipA2 toxin to cope with multiple environmental cues for stress tolerance.

## DISCUSSION

**FIG 7:**
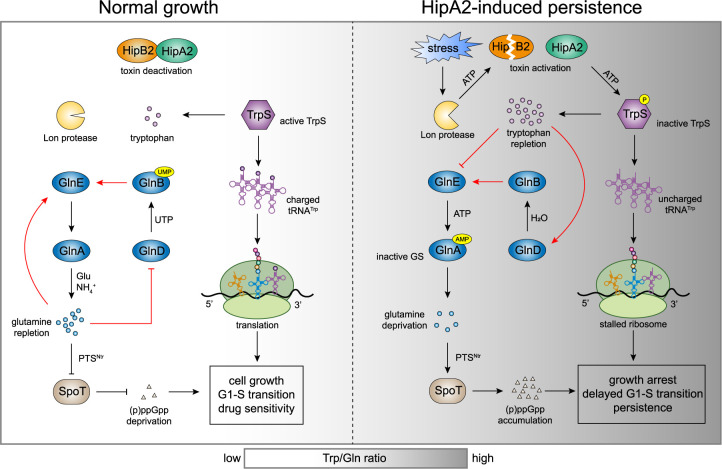
Model of HipA2-induced amino acid imbalance that activates bacterial stringent response by stimulating GlnA (GS) adenylylation. Multiple stresses trigger the liberation of HipA2 toxin by activating Lon protease, which in turn mediates the proteolysis of antitoxin HipB2. The released HipA2 toxin deactivates tryptophanyl-tRNA synthetase TrpS and inhibits aminoacylation of tRNA^Trp^ by phosphorylation, yielding uncharged tRNA^Trp^ and free tryptophan repletion. The loading of uncharged tRNA^Trp^ at empty ribosomal A sites results in translation termination by stalled ribosomes. The high level of intracellular tryptophan triggers allosteric regulation of GlnD and GlnE by direct binding and thus stimulates GlnA adenylylation. GlnA-AMP is inactive and no longer catalyzes glutamine synthesis, leading to the accumulation of (p)ppGpp upon glutamine deprivation in a PTS^Ntr^-dependent manner. HipA2-mediated toxicity enables *Caulobacter* to utilize the stringent response to cope with multiple stress conditions.

To ensure their survival, bacteria have evolved toxin-antitoxin systems that respond to environmental stresses. The activation of a specific toxin induces the arrest of replication and entry into a physiologically dormant (or persister) state. HipBA toxin-antitoxin modules have been discovered in multiple bacteria (15). Here, we report the molecular mechanism of HipA2-induced persistence in the alphaproteobacterium Caulobacter crescentus (Fig. 7). HipA2 encodes a eukaryote-like serine-threonine kinase that phosphorylates tryptophanyl-tRNA synthetase (TrpS) on serine 210, which in turn deactivates the enzyme and inhibits tRNA^Trp^ aminoacylation (Fig. 2). The phosphorylation of TrpS, as well as glutamyl-tRNA synthetase (GltX), was found in the gammaproteobacterium E. coli (8, 9, 38), implying that inhibiting tRNA synthetase activity is a conserved biochemical trait of HipA-family toxins. However, the signaling pathway that transduces the HipA-mediated toxicity to a cellular response can differ, as we demonstrate here. In the case of E. coli, the response pathway is mediated by the stringent response that is controlled by two enzymes, the (p)ppGpp synthetase RelA and the bifunctional (p)ppGpp synthetase/hydrolase SpoT (39, 40). The kinase activity of HipA that inhibits tRNA synthetase activity, in turn, induces the accumulation of (p)ppGpp due to RelA activation by stalled ribosomes upon the binding of uncharged tRNA (8, 9). In contrast, *Caulobacter* encodes a single SpoT enzyme whose activation is dependent on a nitrogen-related phosphotransferase system (22). The binding of uncharged tRNA to the ribosome A site is not sufficient for full activation of SpoT (20). Therefore, the accumulation of uncharged tRNA^Trp^ that halts protein synthesis and increased levels of free intracellular tryptophan that activates stringent response are both required for HipA2-mediated persistence in *Caulobacter*.

*Caulobacter* encodes three HipBA modules on its chromosome. To determine whether encoding more than one copy of an HipBA module is a widespread phenomenon in bacteria, we examined sequence data from 98 bacterial species that encode HipBA modules in 11 different phyla. Among all analyzed bacterial species, 55 species in 8 different phyla encode more than one copy of HipBA modules, of which *Polaromonas* sp. encodes six HipBA paralogs in its genome (see Fig. S6A in the supplemental material). HipBA modules are mostly found in *Proteobacteria*, despite a few found in other phyla (Fig. S6A). Our *in silico* analyses indicate a broad range of bacteria harboring multicopy nonconserved HipBA modules, implying that each HipA protein may have a distinct molecular function in terms of its toxicity (Text S1 and Fig. S6).

10.1128/mBio.03020-20.1 TEXT S1 Phylogenetic analysis of HipBA modules in alphaproteobacteria. Download Text S1, DOCX file, 0.01 MB. Copyright © 2021 Zhou et al. 2021 Zhou et al. https://creativecommons.org/licenses/by/4.0/ This content is distributed under the terms of the Creative Commons Attribution 4.0 International license.

10.1128/mBio.03020-20.7 FIG S6 Phylogenetic analyses of HipA toxin in bacteria. (A) The phylogenetic tree of bacterial species that encode one or more HipBA modules on their genome. The tree is constructed based on the genome sequence of each bacterium. Evolutionary distances are not given. Genera are color coded by phyla. The red, green, and blue lines indicate *Proteobacteria*, *Bacteroidetes*, and *Actinobacteria*, respectively. Each black arrow at the top of the species represents one paralog of the HipBA module in the bacteria. (B) Protein similarity matrix shows similarities and identities between HipA variables. Forty-nine HipA homologues from alphaproteobacteria analyzed in panel A are compared with each other using MatGAT software. The percentages of protein similarity and identity of each comparison are plotted. (C) An NJ tree with collapsed branches (bootstrap values >90%) of HipA proteins in alphaproteobacteria encoding >3 copies of HipBA modules (left panel). Bootstrap values (in percent values) are given for the respective nodes. The left number (percent values from 0 to 100) represents the bootstrap factor obtained from the NJ analysis. The right number (percent values given from 0 to 1) is the bootstrap factor obtained from an ML analysis. The proteins are mainly clustered in six groups. The distribution of HipA paralogs within the given bacterial species is shown by colored circles (right panel), which are identical to the colors in the tree. Download FIG S6, TIF file, 1.2 MB. Copyright © 2021 Zhou et al. 2021 Zhou et al. https://creativecommons.org/licenses/by/4.0/ This content is distributed under the terms of the Creative Commons Attribution 4.0 International license.

We established that *Caulobacter* senses the repletion of intracellular tryptophan due to the HipA2 phosphorylation of TrpS which, in turn, inhibits glutamine synthesis by stimulating GlnA adenylylation (Fig. 4). Why does the cell choose tryptophan? A quantification of absolute metabolite concentrations in E. coli revealed that tryptophan was the least abundant amino acid in the cell (41), which might represent a general fact in bacteria. Manipulating the intracellular levels of a less abundant amino acid might be easier to achieve and does not require a higher energy cost. The accumulation of tryptophan due to the disrupted aminoacylation of tRNA^Trp^ is likely due to the insensitivity of *trp* operon repressor protein to increased levels of free tryptophan in *Caulobacter* (42). It is known that in E. coli tryptophan synthesis is regulated by a *trp* operon, which is turned on for tryptophan synthesis when tryptophan levels are low and turned off when tryptophan levels are high (43). This feedback regulation is mediated by a *trp* repressor protein that binds the promoter region of the *trp* operon to block its transcription when bound to tryptophan (44). However, in *Caulobacter*, the expression of the *trp* operon does not vary significantly in response to tryptophan added to the growth medium (42). Exogenous tryptophan has been shown to inhibit *Caulobacter* growth (45). Furthermore, *Caulobacter* encodes an incomplete tryptophan degradation pathway, often referred to as the kynurenine pathway that consists of three enzymes in E. coli (46). *Caulobacter* has only the first enzyme in the pathway, the tryptophan 2,3-dioxygenase, and the degradation pathway has not been characterized. Therefore, the sink of tryptophan is limited only by its consumption during protein synthesis, which requires TrpS activity. Because TrpS is deactivated by HipA2, we propose that as the level of free intracellular tryptophan increases, it allosterically activates glutamine synthetase adenylyltransferase GlnE, which in turn abolishes glutamine synthesis by GlnA via adenylylation (Fig. 4). By serving as a central nitrogen donor for the biosynthesis of other amino acids, the rapid consumption of glutamine leads to deprivation of such amino acids and, consequently, engenders a stringent response.

HipA3 is likely a noncanonical HipA family toxin because (i) none of the potential targets from our mass spectrometry analysis shows positive result in the Phos-tag SDS-PAGE gel, by which the HipA2 target is successfully verified (Fig. 2A and Fig. S2E); (ii) HipA3 does not have autophosphorylation activity compared to HipA1 and HipA2 (Fig. S2F); and (iii) HipA3 does not have conserved palindromic operator sites in the promoter region, whereas both HipA1 and HipA2 contain three operator sites in their promoter regions (Fig. S2G). Thus, HipA3 may have a distinct mechanism of toxin activity.

We sought to investigate the environmental cues or stress groups specifically sensed by three different HipBA modules in *Caulobacter*. We found that both HipA2 and HipA3 are responsive to only a small subset of stress conditions, which are mostly nutrient starvations or physical stresses such as heat shock and UV exposure (Fig. 6A). Surprisingly, HipA1 appeared to be activated by most of the tested stress conditions. Given that we were unable to detect HipA1-induced growth arrest (Fig. 1C and Fig. S1A), we speculate that HipA1 evolved in a manner that reduced its effect on cell viability so that the cell is less easily perturbed by stochastically activated toxins. It was recently reported that the three HipAs in *Caulobacter* are all functional as toxins when they are individually expressed in the cell without the cognate antitoxin (25). In our experiments, where HipA1 appeared unable to function as a toxin, we ruled out possible suppressor mutations affecting HipA1 function by whole-genome sequencing (see the supplemental material). It may be that a toxic effect of HipA1 induction by an isopropyl-β-d-thiogalactopyranoside (IPTG)- and theophylline-inducible system used in the study by Huang et al. (25) provided a larger amount of protein expression, leading to proteotoxicity by unfolded, insoluble protein aggregates, as we encountered when purifying HipA toxins from E. coli BL21 strains. Most of our antibiotic treatments did not induce upregulation of the HipAs in *Caulobacter* (Fig. 6A). This might reflect the freshwater ecological niche of Caulobacter crescentus, where it is unlikely to face lethal amounts of antibiotics compared to the host milieu faced by pathogenic bacteria.

## MATERIALS AND METHODS

### Bacterial strains, plasmids, and growth conditions.

Bacterial strains and plasmids used in this study are listed in Table S3 in the supplemental material. *Caulobacter* strains were grown in PYE (rich medium) or M2G (minimal medium) at 30°C, supplemented with 0.2% glucose (PYEG), 0.3% xylose (PYEX), or 0.5 mM vanillate (PYEV) when necessary. Antibiotics were supplemented as needed for solid and liquid media, respectively, with the following concentrations: kanamycin (25 μg ml^−1^ or 5 μg ml^−1^), spectinomycin (50 μg ml^−1^ or 25 μg ml^−1^), gentamicin (10 μg ml^−1^ or 5 μg ml^−1^), or chloramphenicol (1 μg ml^−1^ or 2 μg ml^−1^). For bacterial growth curve, optical density was measured at 600 nm (OD_600_) by a 96-well plate reader.

10.1128/mBio.03020-20.10 TABLE S3 Strains and primers used in this study. Download Table S3, XLSX file, 0.01 MB. Copyright © 2021 Zhou et al. 2021 Zhou et al. https://creativecommons.org/licenses/by/4.0/ This content is distributed under the terms of the Creative Commons Attribution 4.0 International license.

### Persistence assays.

Persisters were detected as previously described (47). Biphasic killing curves are often used to distinguish regular cells that are rapidly killed by bactericidal antibiotics and persister cells that are slowly killed. We determined in this study that kanamycin (5 μg/ml) and gentamicin (1 μg/ml) are both suitable bactericidal antibiotics to generate a biphasic killing curve of *Caulobacter* cells and that the persister fraction can be viewed after 4 h post-antibiotic treatment. To quantify persister formation, single colonies were inoculated into PYE or M2G medium unless otherwise noted. Overnight cultures were subdiluted at 1:100 back into PYE or M2G medium and agitated at 30°C in a water bath shaker until mid-exponential phase (OD_600_ of 0.4 to 0.6). CFU counts per milliliter of the initial cultures were determined by plating serial dilutions on PYE agar plates. To eliminate the regular cells, lethal concentrations of the antibiotic kanamycin (5 μg/ml) or gentamicin (1 μg/ml) were added. After 4 h antibiotic treatment, cells were harvested, washed in phosphate-buffered saline (PBS), resuspended in 100 μl of sterile PBS, and serially diluted in PBS. CFU counts per milliliter of the persister cells were determined by plating serial dilutions on PYE agar plates. The plates were incubated at 30°C for 3 days until 10 to 100 bacterial colonies were seen from a certain spot. The fraction of persister cells was calculated as the ratio of the CFU counts per milliliter after and before antibiotic treatment.

### Phosphoproteome analysis. (i) Sample preparation for LC-MS.

A schematic view of experimental design is shown in Fig. S2d. A *hipA* gene of interest was inserted into the *xylX* locus in the *ΔhipBA1BA2BA3* background as a single chromosome copy, providing the xylose-inducible control of toxin expression. A similar strain harboring an empty vector at the same locus serves as a negative control to rule out the HipA-independent phosphorylation. Overnight cultures were diluted at 1:100 in fresh PYE medium supplemented with glucose and grown to exponential phase. Cells were pelleted, washed twice with PYE medium, and resuspended in PYE medium supplemented with xylose to induce the expression of the toxin gene for 3 h. After xylose induction, each sample was pelleted, resuspended in 1 ml of 50 mM Tris-Cl buffer (pH 7.5) with added phosphatase inhibitor cocktail 3 (Millipore-Sigma), incubated 20 min at 37°C, and sonicated three times periodically during incubation with a sonicator probe. Samples were centrifuged at 16,000 × *g* for 10 min to clear the debris, and the supernatant was collected. Protein concentrations were measured with bicinchoninic acid (BCA) assay. Samples were dried in a vacuum centrifuge, denatured, and reduced by resuspending the proteins in 100 μl of 8 M urea with 5 mM dithiothreitol (DTT) in 50 mM triethylammonium bicarbonate (TEAB) buffer at 37°C for 30 min. Samples were alkylated by incubating with 15 mM iodoacetic acid (IAA) in the dark at room temperature. Samples were diluted 10-fold with 50 mM triethylammonium bicarbonate and digested with Lys-C (Wako) at a 1:100 (wt/wt) enzyme-to-protein ratio for 3 h at 37°C. Trypsin was added to a final 1:50 (wt/wt) enzyme-to-protein ratio for overnight digestion at 37°C. Samples were desalted using 100 mg Sep-Pak C_18_ columns (Waters) according to manufacturer’s instructions. The eluted peptides were dried in a vacuum centrifuge, and the phosphopeptides were enriched using the PolyMAC-Ti kit (Tymora Analytical) per manufacturer’s instructions. The eluted phosphopeptide samples were dried completely in a vacuum centrifuge and stored at −80°C.

### (ii) LC-MS/MS analysis.

Dried phosphopeptide samples were dissolved in 4.8 μl of 0.25% formic acid with 3% (vol/vol) acetonitrile, and 4 μl of each was injected into an Easy-nLC 1000 (Thermo Fisher Scientific). Peptides were separated on a 45-cm in-house packed column (360 μm outside diameter [o.d.] × 75 μm inside diameter [i.d.]) containing C_18_ resin (2.2 μm, 100 Å; Michrom Bioresources). The mobile phase buffer consisted of 0.1% formic acid in ultrapure water (buffer A) with an eluting buffer of 0.1% formic acid in 80% (vol/vol) acetonitrile (buffer B) run with a linear 60-min gradient of 6 to 30% buffer B at a flow rate of 250 nl/min. The Easy-nLC 1000 was coupled online with a hybrid high-resolution LTQ-Orbitrap Velos Pro mass spectrometer (Thermo Fisher Scientific). The mass spectrometer was operated in the data-dependent mode, in which a full-scan MS (from *m/z* 300 to 1,500 with the resolution of 30,000 at *m/z* 400) was followed by tandem mass spectrometry (MS/MS) of the 10 most intense ions (normalized collision energy, 30%; automatic gain control [AGC], 3E4; maximum injection time, 100 ms; 90 s exclusion).

### (iii) LC-MS data processing.

The raw files were searched directly against the UniProt Caulobacter crescentus database with no redundant entries, using the Byonic search engine (Protein Metrics) loaded into Proteome Discoverer 2.2 software (Thermo Fisher Scientific). Initial precursor mass tolerance was set at 10 ppm, the final tolerance was set at 6 ppm, and ion trap mass spectrometry (ITMS) MS/MS tolerance was set at 0.6 Da. Search criteria included a static carbamidomethylation of cysteines (+57.0214 Da), and variable modifications of oxidation (+15.9949 Da) on methionine residues, acetylation (+42.011 Da) at the N terminus of proteins, and phosphorylation (+79.996 Da) on serine, threonine, or tyrosine residues were searched. Search was performed with full trypsin/P digestion and allowed a maximum of two missed cleavages on the peptides analyzed from the sequence database. The false-discovery rates of proteins, peptides, and phosphosites were set at 0.01. All protein and peptide identifications were grouped, and any redundant entries were removed. Only unique phosphopeptides and unique master proteins were reported.

### Phosphoprotein detection by Phos-tag SDS-PAGE.

To detect protein phosphorylation by HipAs, the HA-tagged protein of interest and HipA toxin were coexpressed under the control of native promoter and xylose promoter, respectively. Strains were grown in PYE supplemented with glucose to exponential phase. Cells were washed with fresh PYE and resuspended in PYE supplemented with xylose to induce toxin expression for 3 h. Samples before induction and after induction were normalized to a similar cell density and lysed in Laemmli sample buffer. Protein samples were subjected to phosphate-affinity SDS-PAGE with acrylamide-pendant Phos tag (Wako) in accordance with the manufacturer’s instructions and immunoblotted with anti-HA antibody.

### Protein purification.

For purification of HipA toxin, the full-length open reading frame (ORF) of *hipA* was cloned into pMAL-c5x. The Q5 site-directed mutagenesis kit was used to generate a kinase-dead mutant. The recombinant plasmids were introduced into ER2566. A 500-ml culture of E. coli cells was grown at 37°C in LB medium (with the addition of 0.2%) to an optical density at 600 nm of 0.6 and then incubated with 1 mM isopropyl-β-d-thiogalactopyranoside (IPTG) for 3 h. Cells were sonicated in purification buffer (25 mM HEPES [pH 8.0], 200 mM NaCl, 1 mM EDTA, 1 mM DTT). Cell lysate was incubated with amylose resin for 20 min. The amylose column was washed four times with 10 volumes of purification buffer. Protein was eluted in purification buffer supplied with 10 mM maltose and 20% glycerol. Control MBP was purified by the same procedure. For purification of histidine-tagged TrpS and its variant, crude extracts from E. coli cells were incubated with Ni-NTA resin for 30 min. The agarose beads were then washed with 10 volumes of purification buffer with the addition of 20 mM imidazole. The protein was eluted in purification buffer containing 300 mM imidazole. MBP-tagged HipA proteins were further polished by gel filtration (Sephacryl S-200 column). All proteins were dialyzed against purification buffer supplied with 30% glycerol and stored at −20°C. GlnE and GlnD were purified similarly to TrpS.

### *In vitro* kinase assay.

TrpS or TrpS210A (2 μM) was mixed in kinase reaction buffer (25 mM Tris-HCl, pH 7.5, 10 mM MgCl_2_, 10 mM MnCl_2_, 20 μM ATP, 0.5 mM DTT) with 0.2 μM HipA2, 4 μCi/μl [γ-^32^P]ATP. The reaction mixture was incubated at 30°C for 1 h, and the reaction was stopped by the addition of 1 volume of Laemmli buffer, resolved by SDS-PAGE, and revealed by phosphorimaging (GE Healthcare).

### GlnA pulldown assay.

A strain expressing HA-GlnA under the control of its native promoter and HipA2 driven by the xylose promoter was grown overnight in PYEG, subdiluted in fresh PYEG at 1:100, and regrown to exponential phase. Cells were washed twice with PYE, reinoculated in PYEG or PYEX, and agitated at 30°C for 3 h. Samples were harvested and adjusted to an OD_600_ of 1 in 30 ml PYE so that each sample contained a similar number of cells. Cells were pelleted, washed once with cold PBS, and resuspended in 500 μl PBS supplemented with protease inhibitor cocktail and 1 mM DTT. Cells were lysed by sonication, and cell lysates were mixed with 80 μl anti-HA magnetic beads (Thermo Scientific Pierce). The immunoprecipitation is performed according to manufacturer’s instructions. HA-GlnA was eluted with Pierce HA peptide and detected by gel immunoblotting.

### Microscopy.

C. crescentus strains were grown in PYEG to exponential phase (OD_600_ < 0.3) at 30°C. Cells were washed and recultured in PYEX to induce toxin expression for 3 h. Cells were then exposed to kanamycin (5 μg ml^−1^) for an additional 4 h. Cells were pelleted, washed twice with distilled water, and stained with SYTOX Deep Red nucleic acid stain in accordance with the manufacturer’s instructions. Phase-contrast and fluorescence microscopy images were obtained using a Leica DMi8 microscope with an HC PL APO 100×/1.40 oil PH3 objective, Hamamatsu electron-multiplying charge-coupled device (EMCCD) C9100 camera, and Leica Application Suite X software. For all image panels, the brightness and contrast of the images were balanced with ImageJ (NIH) to represent foci or diffuse fluorescent signal. For computational image analyses, MicrobeJ (48) was used to determine fluorescence intensities of YFP and SYTOX Deep Red. The data were plotted and statistically analyzed using Prism 7 (GraphPad).

### Quantification of intracellular (p)ppGpp accumulation.

The quantification of intracellular (p)ppGpp accumulation was performed as previously described (22) with modifications. Briefly, *Caulobacter* cells were grown in PYE medium overnight. Cultures were subdiluted in M5GG (supplemented with 1 mM glutamate) and grown overnight. Cells were subdiluted for a second time in M5GG and grown to an OD_600_ of 0.4. Vanillate or water (as a control) was added to the culture to induce toxin gene expression for an additional 1 h. A 1.0-ml aliquot was removed from each culture, pelleted at 16,000 × *g*, washed two times in M5GG, and resuspended in 225 μl M5GG. For carbon starvation, the aliquot was washed and resuspended in M5G without glucose. KH_2_^32^PO_4_ was added at 100 μCi/ml, and cultures were labeled for 2 h with shaking (200 rpm) at 30°C. Following the labeling, cells were pelleted at 16,000 × *g*, washed one time with M5GG, resuspended in 225 μl M5GG, and mixed with an equal volume of 2 M formic acid for (p)ppGpp extraction. Samples were placed on ice for 30 min and stored at −20°C overnight. For visualizing (p)ppGpp levels, cell extracts were pelleted at 16,000 × *g* for 3 min and 3 × 2 μl of supernatant was spotted onto a polyethyleneimine plate (Sigma). Polyethyleneimine plates were then developed in 1.5 M KH_2_PO_4_ (pH 3.4) at room temperature (RT). TLC plates were imaged on a phosphor screen (GE Healthcare) and analyzed with a Typhoon storage phosphorimager (Molecular Dynamics). Band intensities were quantified using ImageJ.

### Quantification of intracellular free amino acid.

Cells were grown in 100 ml M2G medium to the exponential phase (OD_600_ = 0.5). HipA2 toxin was induced for 3 h by adding vanillate to a final concentration of 0.5 mM. Twenty milliliters of culture was withdrawn at each hour upon induction for quantification of intracellular free amino acid. Cells were pelleted by centrifugation, washed once with cold PBS buffer, and resuspended in 300 μl of distilled water. The cell suspensions were lysed by sonication. The clear lysates were diluted using 700 μl absolute (100%) ethanol to a final concentration of 70% ethanol and incubated at −20°C for 30 min. A final centrifuge removed the resulting precipitate. The clarified supernatants containing the ethanol-extracted amino acid were freeze-dried, and the lyophilized samples were resuspended in 20 μl cold distilled water for free amino acid quantification. Free tryptophan and glutamine levels were assayed using the Bridge-It tryptophan fluorescence assay (Mediomics) and Glutamine/Glutamate-Glo Assay (Promega) according to the manufacturer’s protocol, respectively.

### Surface plasmon resonance.

The interactions of amino acids (tryptophan and glutamine) with GlnD and GlnE proteins were analyzed by SPR using a Biacore T200 biosensor system (GE Healthcare). Amino acid dilutions were performed in running buffer (25 mM HEPES at pH 8, 200 mM NaCl). GlnD or GlnE protein was dialyzed against running buffer overnight and immobilized onto a CM5 biosensor chip by carboxymethylated dextran with covalently immobilized Ni-NTA. The dilutions of amino acids were injected over the immobilized protein surfaces. The experiments were performed at 25°C using a flow rate of 50 μl min^−1^. For each experiment, at least 5 different concentrations of amino acid were injected over each experimental and control flow cell for 60 s. Dissociation was allowed to occur at the same flow rate for 600 s in running buffer alone. All data were corrected for nonspecific binding by subtracting the signal measured in a control cell lacking immobilized ligand. Both data processing and kinetic fitting were performed using BIAevaluation software v2.0 (GE Healthcare).

### Protein *in vivo* stability assay.

The HipB *in vivo* stabilities were assayed as described previously (49). Briefly, strains harboring the His-*hipBA* operon with its native promoter on a replicating plasmid were grown in PYE to exponential phase. Indicated stresses were applied to the culture for 30 min. Protein synthesis was shut off immediately by the addition of 200 μg/ml chloramphenicol. Samples were taken at the time points as indicated in the figurse and snap-frozen in liquid nitrogen before immunoblot analysis.

### qRT-PCR.

*Caulobacter* wild-type cells were cultured in PYE to exponential phase (OD_600_ ≈ 0.4). Indicated stress was applied to the culture for 30 min (see Table S2 for detailed information). Cells were harvested, treated with 2 volumes of RNAprotect bacterial reagent (Qiagen), and snap-frozen in liquid nitrogen. The total RNA was extracted using the Qiagen RNeasy minikit. Contaminated genomic DNA was removed through on-column digestion with DNase using the Qiagen RNase-free DNase kit. The RNA concentration was determined using a NanoDrop 2000 spectrophotometer (Thermo Scientific). Reverse transcription and cDNA synthesis were performed using the QuantiTect reverse transcription kit. Quantitative PCRs were performed using Luna universal qPCR master mix (NEB) on an Applied Biosystems 7500 Fast real-time PCR system. The *gyrB* gene was used as an endogenous control. The relative fold change in target gene expression was calculated using a threshold cycle (2^–ΔΔ^*^CT^*) method.

### Statistical analysis and data availability.

Prism GraphPad was used to perform statistical analyses. Information regarding individual statistical test parameters can be found in the figure legends. Genome resequencing data of HipA1 suppressors can be found on the Sequence Read Archive (accession number PRJNA670525).
