# Supplementary material for: A Bacterial Toxin Perturbs Intracellular Amino Acid Balance To Induce Persistence
Source: mBio. 2021 Feb 23;12(1):e03020-20. doi: 10.1128/mBio.03020-20 (PMC8545095; doi:10.1128/mBio.03020-20)
Supplement: TEXT S1 [file mbio.03020-20-s0001.docx]

# Supplementary document

## Phylogenetic analysis of HipBA modules in alpha-proteobacteria

In alpha-proteobacteria, our analysis included 27 species encoding 52 HipBA homologs in total, of which 14 species encoded multiple HipBA modules. We compared protein identity and similarity between each HipA toxin homolog in all analyzed alpha-proteobacteria and found that HipA toxins are poorly conserved between homologs (Supplementary Fig. 6B). Similar results were also observed for HipA paralogs within a bacterial species. To gain evolutionary insights into HipA toxins, we built HipA protein phylogenetic trees using both the neighbor-joining (BIONJ) method and the maximum-likelihood (ML) method. The unrooted BIONJ tree with collapsed branches (node support value >90%) is depicted in Supplementary Fig. 6C. This phylogenetic analysis revealed that 52 HipA toxins clustered in six main clades (Supplementary Fig. 6C). Clades I, III, V and VI were relatively larger than clades II and IV (Supplementary Fig. 6C). Notably, different HipA paralogs encoded by the same bacterial species were often not clustered in the same clade, which is consistent with our protein similarity data (Supplementary Fig. 6B). The distributions of HipA paralogs to each cluster were analyzed in 8 bacterial species that encoded multiple HipBA modules on their genomes. Results showed no direct linkage between evolutionary relationship and clustered distribution of HipA toxins (Supplementary Fig. 6C).
