# Supplementary figures and images for: A Bacterial Toxin Perturbs Intracellular Amino Acid Balance To Induce Persistence
Source: mBio. 2021 Feb 23;12(1):e03020-20. doi: 10.1128/mBio.03020-20 (PMC8545095; doi:10.1128/mBio.03020-20)

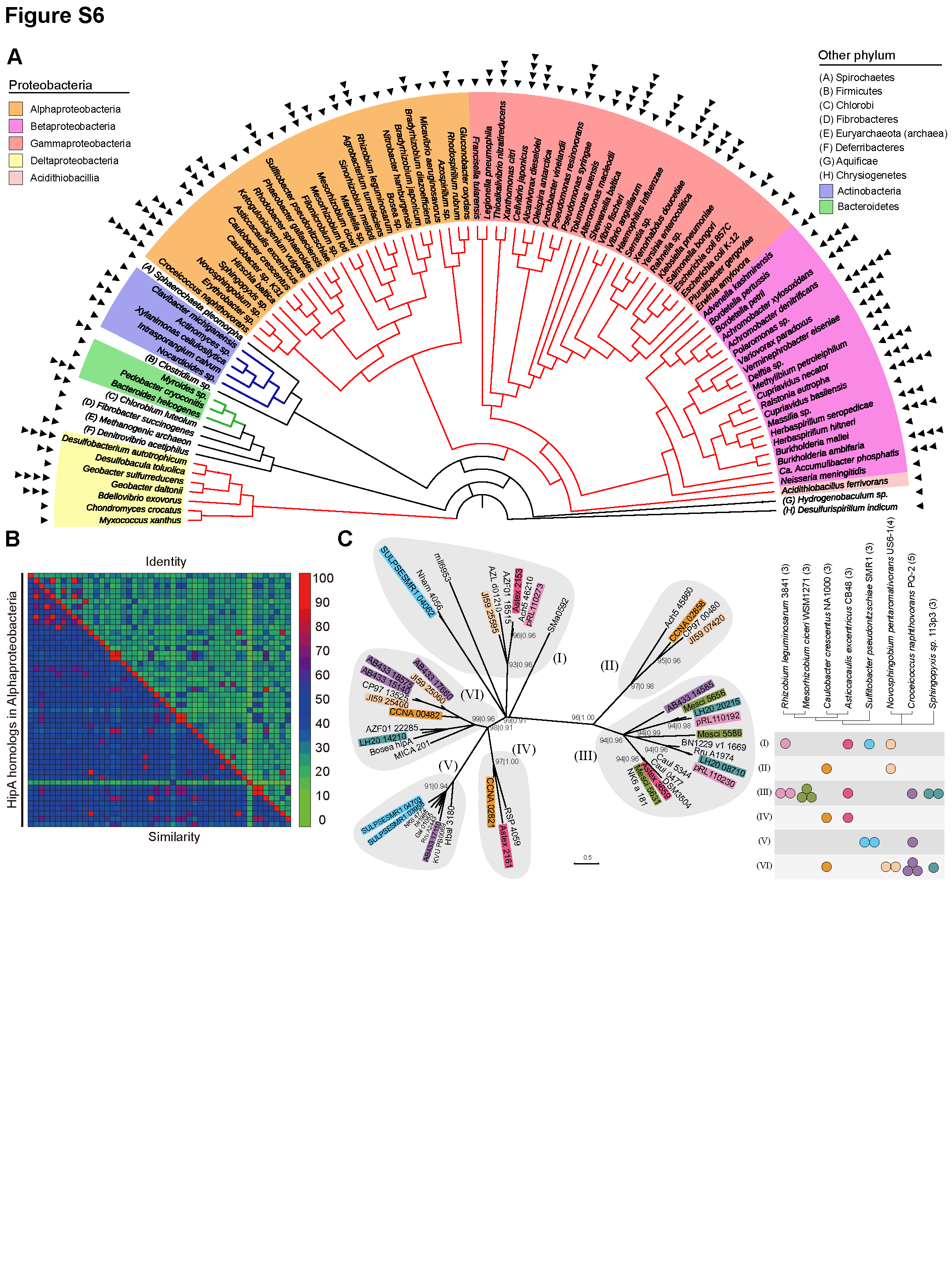

Supplement: FIG S6 [file mbio.03020-20-sf006.tif]
